# Role of inflammation in atrial fibrillation: A comprehensive review of current knowledge

**DOI:** 10.1002/joa3.12473

**Published:** 2020-12-23

**Authors:** Nso Nso, Kaveh R. Bookani, Mark Metzl, Farshid Radparvar

**Affiliations:** ^1^ Department of Internal Medicine Division of Cardiology Icahn School of Medicine at Mount Sinai/Queens (Queens Hospital Center) Jamaica NY USA; ^2^ Department of Medicine Division of Cardiovascular Medicine (Northshore Program) University of Chicago Evanston IL USA

**Keywords:** atrial fibrillation, cardiac arrhythmia, inflammatory biomarkers

## Abstract

**Background:**

Atrial fibrillation (AF) is one of the most common cardiac disorders affecting adults and is associated with significant morbidity and mortality. Efforts to manage AF through anti‐arrhythmics and rate control have been largely unsatisfactory. It has become clear that AF causes structural alterations in the atrial myocardium that propagate further AF, and that some of these alterations are the result of inflammation.

**Methods:**

An in‐depth review of the available literature was undertaken using Google Scholar and keyword searches including [Atrial fibrillation] in combination with [inflammatory markers], [myocardial fibrosis], and [immunomodulators], limiting the search to English language articles. All articles were reviewed for relevance and collated by the author.

**Results:**

Multiple markers of inflammation have been shown to be elevated in AF and to predict responses to treatments of AF including anti‐arrhythmics and cardioversion. The nidus of inflammation is not clear but seems to be related to the pulmonary veins.

**Conclusions:**

The inflammatory cascade induces fibrotic changes in the myocardium, an arrhythmogenic process that stimulates further inflammation. Advances in treatment are focusing on biological agents and immunomodulators that inhibit the inflammatory cascade.

## Graphical abstract

Our paper describes the most current understanding of the role of inflammation in the pathophysiology of atrial fibrillation. Current science has shown that atrial fibrillation causes inflammation and structural changes in the myocardium, and that inflammation and the changes in structure work together to reduce the threshold for atrial fibrillation. The mediators of this process are delineated in the paper along with recommendations for future research and treatment.

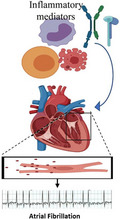

## 1 INTRODUCTION

Atrial fibrillation (AF) is the most common clinically relevant cardiac arrhythmia in adults. It is more common in the elderly, affecting about 1% of people under 65 years old but 5% of people over 65.^1^ AF is associated with significant morbidity and mortality and has adverse effects on overall quality of life. Even patients with short‐term (≤7 days) AF have an increased risk of stroke.^2^ Furthermore, given the aging of our society, the economic burden of AF is enormous.

Current management options are suboptimal. Outcomes of rhythm and rate control efforts have shown the limited efficacy and high side effects of both methods.^3^ Therefore, there is a significant need for alternative, safe, and effective methods of treatment. This strong need has prompted a fundamental re‐evaluation of the pathophysiology of AF with the goal of finding new approaches to treatment.

Inflammatory processes are among the major focuses of current medical research and there are increasing evidences that inflammation may play a major role in cardiovascular diseases including myocardial disease, atherosclerotic disease, and strokes.^4^ The role of inflammation in AF is becoming more clear, changing the paradigm from one of an electrical abnormality to a biochemical, structural disorder. Previously Wu et al^5^, ^6^ have compiled list of studies reporting role of inflammation in the perpetuation of AF. Guadino et al^7^ linked an inflammatory marker (Interleukin‐6 [IL‐6]) polymorphism with postoperative AF while C‐reactive protein has been implicated in AF both post‐^8^ and non‐postoperative patients.^9^ By means of multivariable proportional hazards model, Schnabel et al reported association of 12 inflammatory markers with AF incidence.^10^ Therapies focused at reducing the inflammatory affliction are encouraging for AF treatment. Chokesuwattanaskul et al have compiled a list of studies linking NSAIDs with AF reduction.^11^ The aim of this article is to describe the role of different inflammatory markers in AF.

## 2 WHAT IS THE PATHOPHYSIOLOGY OF AF?

**FIGURE 1:**
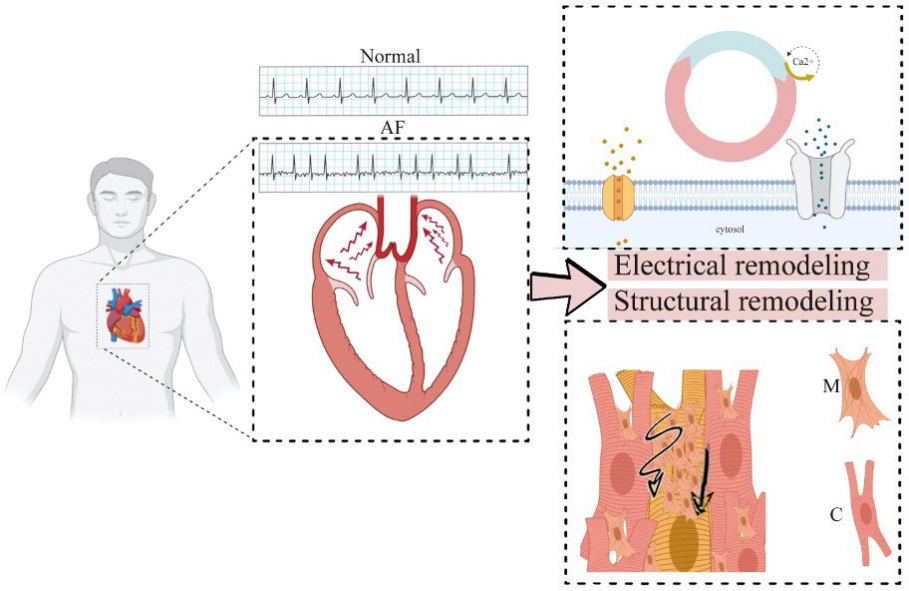
Major changes caused by atrial fibrillation (AF) include electrical and structural remodeling. Minute but irregular changes in electrocardiogram of AF patient can be seen in the figure. Ca^2+^ is the center of the electrical changes, with several more perturbations linked to it. Increased calcium leads to short refractory period (depicted by blue in the cycle), leading to transmission of re‐entrant waves, breaking normal sequence, and setting up more cycles. At tissue scale structural remodeling, conduction is slowed by the myofibroblast (represented by M) secreting collagen (shown in yellow), leading to fibrosis. Myofibroblast is involved in ionic remodeling and its interaction with cardiomyocyte (represented by C) also slackens conduction

Atrial fibrillation, once established, causes changes in the atrial myocardium which are known as “cardiac remodeling” (Figure 1). Electrical remodeling in AF promotes ongoing re‐entrant depolarizations that cause more AF.^12^ The atrial refractory period shortens and atrial conductivity lengthens, while calcium accumulation in atrial myocytes further shortens the refractory period. Multiple small re‐entrant circuits develop.^13^ It has also been reported that reactive oxygen species from mitochondria activate CAMKII, leading to the phosphorylation of another marker RYR2. This initiates AF, as observed in mouse model studies. Oxidative stress heightens AF and triggers the marker CAMKII to associate Ca^2+^ with calmodulin and stimulate it.^14^ The adage “AF begets AF” applies.

There are structural remodeling changes as well, including left atrial dilatation and atrial fibrosis. The intercellular fibrosis impedes atrial conduction, affecting the biophysical and electrical properties of the atrial wall.^15^ The loss of signal coordination and control also adds to the perpetuation of AF.

Collagen‐based tissue fibrosis is important as a reparative process after tissue damage or cell death. Normal atrial myocytes are stacked end‐to‐end, connected by specialized intercellular proteins called connexins. The connexins work in the gap junctions, forming low‐resistance communications channels between cells.^15^ In the myocardium, apoptotic or necrotic cells are replaced by collagen following infarction or exposure to toxins, so‐called reparative fibrosis, replacing individual cells within a stack of myocytes. This results in a separation of cells from end‐to‐end, interfering with intercellular communication through endplates. Reactive fibrosis occurs in response to inflammation, tachycardia, and altered clearing of collagen. This is most commonly seen in hypertension and diabetes mellitus, through effects of the renin‐angiotensin aldosterone system, beta‐adrenergic system, excessive reactive oxygen species, and metabolic disturbances caused by hyperglycemia.^16^, ^17^ Collagen fibers are deposited between strands of myocyte stacks, increasing the volume of intercellular matrix and altering communication between bundles of myocytes.^15^ While reactive fibrosis begins early, eventually individual myocytes become necrotic and are replaced by reparative fibrosis.^18^ Since reparative fibrosis directly impacts on connexins, it is felt to be a more significant problem in chronic atrial fibrillation.

At a molecular level, fibrosis is mediated by several signaling pathways including angiotensin II, transforming growth factor (TGF)‐beta 1, platelet‐derived growth factor (PDGF), and connective tissue growth factor. TGF‐β1 works in concert with a transmembrane protein called CD44 that stimulates cell's STAT3 pathway, increasing expression of hyaluronic acid (HA) and collagen. The angiotensin II receptor AT1 stimulates the CD44/HA/STAT3 interaction, promoting fibrosis.^19^ Other mediators have been described in the fibrosis of AF, including oxidative stress marker GDF‐15 linked with bleeding, stroke and death in AF.^20^ Oxidative stress in diabetes and obesity aggravates structural remodeling and hence, AF.^21^

Osteopontin, an extracellular bridging protein, was recently shown to stimulate atrial fibrosis by promoting the proliferation of fibroblasts, production of collagen I, and secretion of fibronectin.^22^ Atrial natriuretic protein, its receptor, and its clearance receptor may also play a role in atrial wall fibrosis contributing to AF.^23^ Spronk et al focused on the effects of atrial fibrillation and a hypercoagulable state.^24^ The findings of their experiments confirmed that, while AF induced a hypercoagulable state, the hypercoagulable state during AF induced profibrotic and proinflammatory responses in atrial fibroblasts. They proposed that the inhibition of coagulation may not only help prevent strokes but may also help prevent the fibrosis seen in AF.

The location of the initial trigger of AF is generally not known, but the pulmonary veins have been implicated in recent research.^25^ Inflammation in the pulmonary vein may act as the nidus for AF, and continued inflammation likely mediates the remodeling process as described above. Studies have identified structural changes within the myocardium that begins without a few hours of onset of AF^26^, consistent with the difficulty of successful restoration of sinus rhythm by cardioversion after 24 hours. This reinforces the concept of structural rather than simply electrical changes.

## 3 IS THERE A CONNECTION BETWEEN INFLAMMATION AND AF?

There is increasing evidence to link inflammation to AF as in several other cardiovascular diseases.^27^ AF is commonly seen as a complication of pericarditis, myocarditis, and endocarditis. The earliest reference to the idea that AF and inflammation were connected was posed by Bruins after the observation that AF occurred on the second or third day after coronary artery bypass surgery at about the same time the C‐reactive protein (CRP) levels peaked.^28^ The following year, a large percentage of patients with intermittent AF of unknown etiology were noted to have antibodies against cardiac myofibril components.^29^ These two studies supported the idea that AF was an inflammatory condition.

## 4 WHAT IS THE LABORATORY EVIDENCE OF INFLAMMATION IN AF?

**FIGURE 2:**
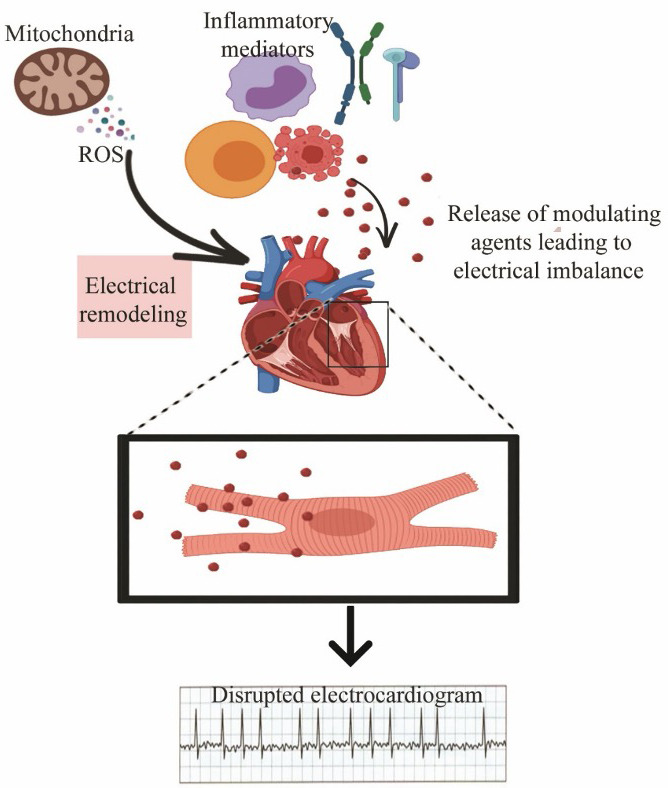
Mitochondrial‐derived reactive oxygen species and inflammatory mediators such as interleukins, cytokines (monocytes shown in yellow, macrophages shown in red, lymphocytes in purple) etc trigger atrial fibrillation

After these two landmark studies were published, investigators started to examine the possible role of variety of inflammatory markers in AF. Although in most of these analyses, it was not clear whether there was a cause‐and‐effect relationship between AF and inflammation, but there has been strong evidence of an association between these conditions (Figure 2). Laboratory studies have revealed basis of higher cardiac inflammation and AF in aged females.^30^ Inflammation association with AF in rat studies has been summarized in Table 1. Postoperative link of inflammation and AF has been established in canine model.^31^ Hamanaka et al further reported in a single center study, that the bleeding risk is elevated in patients with nonvalvular AF.^32^ Choi et al reported association between augmented chance of AF in patients with inflammatory bowel disease.^33^ The two markers of inflammation most often mentioned in relation to AF are high‐sensitivity CRP (hsCRP) and IL‐6. Other reported markers include tumor necrosis factor‐α (TNF‐α), interleukin‐2 (IL‐2), and interleukin‐8 (IL‐8).

**TABLE 1:** Studies reporting AF linkage to inflammatory markers in laboratory rats

Serial no.	Marker	Correlation	Reference
1	TNF, interleukin‐1β and interleukin‐6	Negative	34
2	Interleukin‐10	Positive	34
3	Natriuretic peptide A mutant	Positive	35
4	Collagen 1, Collagen 3, fibronectin, matrix metalloproteinases 2 and 9	Positive	36
5	Interleukin‐17A, IL‐6, IL‐1β and TGF‐β1	Positive	37
6	Col‐1, Col‐3, and α‐SMA	Negative	37
7	IL‐6 and TNF‐α	Positive	38
8	CC chemokine receptor 2+ ED‐1+, ED‐1+ macrophages Interleukin‐6, TNF‐α and nuclear p65	Positive	39

### 4.1 C‐reactive protein

Of the various inflammatory markers, hsCRP has proven to be the most predictable indicator of vascular inflammation. CRP is described as an acute‐phase reactant secreted by the liver in response to IL‐6 production by macrophages and T‐cells. Physiologically, it binds to dead or dying cells and activates the complement system. CRP is synthesized in response to circulating factors released by macrophages and adipocytes, explaining the low‐level inflammatory state that accompanies obesity.^40^ Elevated hsCRP has consistently been associated with the risk of cardiovascular events including myocardial ischemia and infarction, sudden cardiac death, stroke, and peripheral vascular disease.^41^ Patients with AF tend to have a higher level of hsCRP than people in normal sinus rhythm.^8^ Patients in chronic atrial fibrillation have a higher level of hsCRP than people with paroxysmal AF. Finally, people with long‐standing AF tend to show higher levels of hsCRP and more atrial remodeling and dilatation than those with shorter duration AF. After cardioversion, patients with higher hsCRP are more likely to relapse into AF than those with lower serum levels.^42^ Patients in normal sinus rhythm with high levels of hsCRP are more likely to develop AF. These studies emphasize the relationship between inflammation and AF.

The underlying cause of the relationship between hsCRP and AF is still unclear. CRP appears to bind to the membranes of myocardial cells, activating the complement cascade and triggering damage to the tissues.^43^ Immunohistochemical studies have shown high levels of CRP in atheroma in coronary artery disease models.^44^ hsCRP appears to fall in response to statin therapy.^45^

### 4.2 Interleukin‐6

Interleukin‐6 is produced by macrophages, T‐cells, and endothelial cells and has both pro‐inflammatory and anti‐inflammatory functions. It is a cytokine that stimulates the production of CRP, fibrinogen, and serum amyloid‐A. Released after the interaction of immune cells and pathogens, it stimulates the production of several downstream cytokines and triggers fever. In muscle, it is released in response to exercise and downregulates inflammation through its control of tumor necrosis factor TNF‐α.^46^

Although IL‐6 is considered cytoprotective in cardiac and muscle tissues^47^, studies of IL‐6 levels in AF have been mixed. The ARISTOTLE trial of 18 201 patients with AF showed higher mortality in patients with higher IL‐6 levels compared to patients with normal levels.^48^ Another large cohort study, CRIC (Chronic Renal Insufficiency Cohort), found that plasma IL‐6 level is an independent and consistent predictor of AF in patients with chronic kidney disease.^49^ Landiolol, a super‐short‐acting β‐blocker used in Europe and Japan during thoracic surgery to control heart rate, was associated with significantly lower postesophagectomy AF and lower IL‐6 levels.^50^ IL‐6 was shown to rapidly induce electrical remodeling through its effect on intercellular connexins, decreasing cell‐to‐cell communication.^51^ In a large randomized placebo‐controlled trial, the IL‐6 antagonist canakinumab approved for treatment of rheumatologic disorders was shown to reduce cardiovascular events (nonfatal myocardial infarction, nonfatal stroke, and cardiovascular death) vs placebo in a dose‐dependent manner.^52^ That study did not evaluate the impact of canakinumab on atrial fibrillation. Curiously, tocilizumab, a biologic immunosuppressant used in the treatment of rheumatoid disorders, selectively blocks IL‐6 receptors. It is being studied as an anti‐rejection agent in cardiac transplantation,^53^ but it has not been used in the treatment or prevention of AF because of its potentially serious side effects and high cost. IL‐6 may have a role in increasing the prothrombotic state seen in patients with AF.^54^

### 4.3 Tumor necrosis factor

Tumor necrosis factor (TNF), formerly called TNF‐α, is a cell‐signaling protein involved in the inflammatory cascade. Produced by macrophages, CD4+ lymphocytes, NK cells, neutrophils, mast cells, eosinophils, and neurons, TNF is an immune cell activator and a pyrogen, directly triggering fever in animals. It has been shown to cause CRP production by the liver and to stimulate systemic cytokine reactions to infection.^55^

Similar to other inflammatory markers, TNF levels are higher in patients with AF. A study by Hai of 67 patients who were scheduled to have cardiac surgery (31 with rheumatic heart disease and AF, 36 with other heart disease and sinus rhythm) found higher levels of TNF in the AF group compared with the sinus rhythm group.^56^ Multiple animal studies have shown reductions in atrial fibrillation with the use of TNF and TNF‐receptor blockers. A review of the literature by Ren et al showed beneficial effects of TNF blockade in preventing AF.^57^

### 4.4 Interleukin‐2

Interleukin‐2 is another inflammatory cytokine that is produced by activated CD4+ and CD8+ T‐cells. It is involved in the inflammatory cascade and stimulates immune cells, often in response to microbial invaders. It functions through an interaction between the IL‐2 receptor and a cell's JAK/STAT signaling pathway.^58^

Interleukin‐2 has been shown to correlate with the risk of developing AF after cardiac surgery. In one study by Haq, 33 patients undergoing coronary artery bypass graft (CABG) surgery were followed. Eleven developed AF within 1‐2 days postoperatively. Those with AF had a higher IL‐2 level than those without AF, and those who developed AF early (<24 hours) had a higher level of IL‐2 than those who developed AF later.^59^ Rizos et al showed that patients undergoing cardioversion for AF were less often successful if they had higher IL‐2 levels.^60^ Another study showed that IL‐2 level was an independent predictor of recurring AF at the 1‐year follow‐up postpulmonary vein catheter ablation for AF.^61^ Based on our current knowledge, one of the mechanisms of action of lovastatin is inhibition of IL‐2 production with reduction in the level of systemic inflammation in patients with atherosclerosis^62^ with potential therapeutic implications in patients with AF.

### 4.5 Interleukin‐8

Physiologically, IL‐8 recruits inflammatory cells to attack and phagocytize an antigen. Also known as the neutrophil chemotactic factor, it stimulates migration of neutrophils and granulocytes to a tissue, then stimulates phagocytosis once they arrive. IL‐8 increases angiogenesis and triggers local histamine release.^63^

Interleukin‐8 levels rise in the serum of patients with AF.^64^ In a cohort of 113 patients undergoing CABG surgery, patients with postoperative or sustained AF after surgery had significantly higher levels of IL‐8 than patients in sinus rhythm.^6^ A similar study in 2014 found a correlation between inflammatory markers including IL‐8 and the development of AF after CABG surgery.^65^ The origin of the IL‐8 in patients with AF is unclear, but work by Liuba et al suggests that it originates from the peripheral blood stream rather than locally.^66^

### 4.6 White blood cell counts

Leukocytosis, while a non‐specific indicator of inflammation appears to predict AF after cardiac surgery. In a study of 272 patients who underwent lobectomy, pneumonectomy, or esophagectomy, a twofold increase in white blood cell count was associated with a threefold increase in the risk of AF.^67^ A similar study by Lamm et al of 253 patients with normal left ventricular (LF) function undergoing elective cardiac surgery found that leukocytosis was associated with an increased risk of postoperative AF.^68^ Preoperative leukocytosis in CABG predicted AF in a cohort of 66 patients in a study published in 2009.^69^ Leukocytosis is seen as a general indicator of systemic inflammatory and oxidative state, signaling which patients may be at risk for the development of AF.

## 5 IMPLICATIONS FOR PRACTICE

**FIGURE 3:**
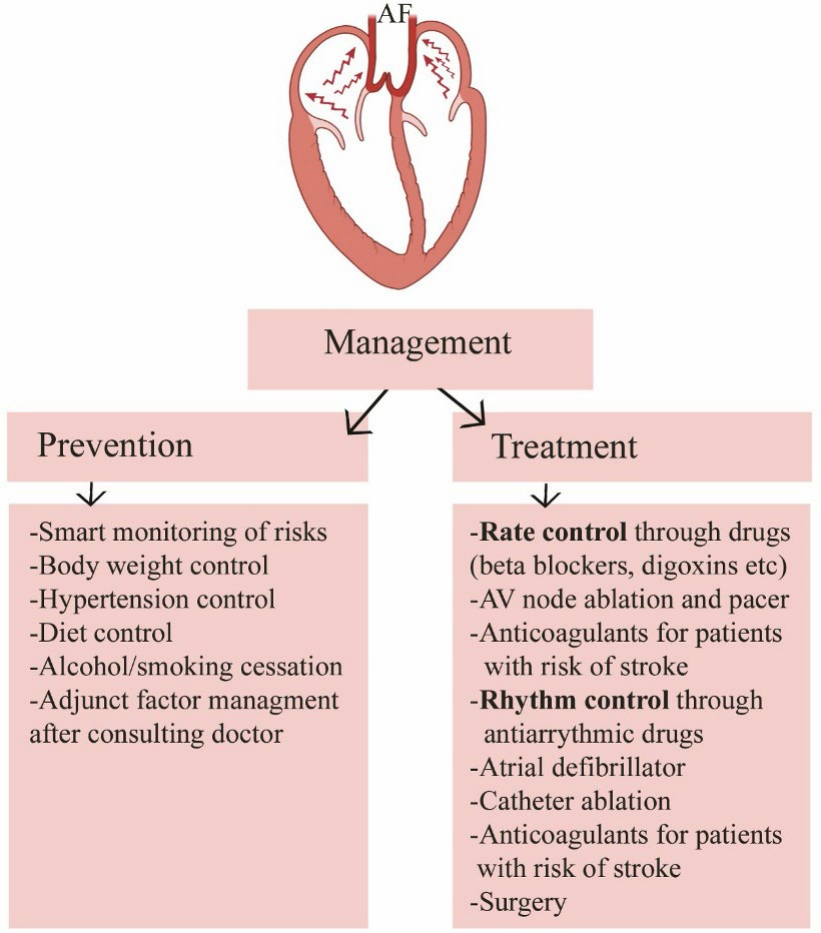
Overview of management of the atrial fibrillation (AF). Both prevention and treatment measures are necessary for successful therapy

Management of the AF requires treatment and prevention strategies (Figure 3). Prevention can be done via adopting healthy life style and reducing the risks while for treatment, several options are available. Given the number of inflammatory markers that are elevated in AF, it is reasonable to classify AF as an inflammatory state. There are still lingering arguments about cause and effect, with some experts arguing that AF causes inflammation instead of the converse. Clearly there is crosstalk as well as other variables such as other hormones and cytokines involved. There have been several classes of medications that have been shown to be helpful in the prevention of AF.

### 5.1 Angiotensin converting enzyme inhibitors (ACEi) and angiotensin receptor blockers (ARBs)

Angiotensin II is a key hormone in regulating blood pressure, vascular smooth muscle tone, renin production, and renal reabsorption of sodium in the distal tubule. Angiotensin II has been found to be pro‐inflammatory, and numerous studies have documented the effectiveness of both ACEi and ARBs in the primary and secondary prevention of AF.^70^ Modulation of the renin‐angiotensin‐aldosterone system (RAAS) reduces the development of atrial fibrosis and cardiac remodeling, key findings in patients with AF. Animal studies have shown the effects of angiotensin II on remodeling, fibrosis, and repolarization in the atria as contributors to the development of AF.^71^ Both ACEi and ARBs appear to be equally effective.^72^

There have been several proposed mechanisms for the effectiveness of ACEi and ARBs in the prevention of AF, including a decrease in atrial stretch, lowered atrial pressures, prevention of angiotensin‐induced fibrosis, reduced sympathetic tone, and direct antiarrhythmic effects.^73^ Furthermore, RAAS modulators likely reduce atrial wall inflammation, oxidative stress, and remodeling.^74^

### 5.2 Statins

Hydroxymethyl glutaryl coenzyme A reductase (HMG CoA) inhibitors are potent lipid lowering agents with secondary anti‐inflammatory properties. These agents have also been shown to enhance the function of endothelial cells, increase nitric oxide, reduce thrombosis, stabilize plaques, and reduce oxidative stress.^75^

More recently, statins' anti‐arrhythmic properties have been explored in relation to AF. In fact, they have been shown to be effective in primary and secondary prevention of AF. Siu et al studied 62 patients with chronic AF undergoing successful cardioversion, and showed significant reductions in recurrence of AF with the use of statins.^76^ A study by Young‐Xiu evaluated a cohort of 499 patients with coronary artery disease and found that those taking statins were significantly less likely to develop AF.^77^ Studies by Reilly et al suggested that statins are more effective early in the course of AF rather than later as a management strategy of chronic AF.^78^ The combination of ACEi and statins was shown to be highly effective at reducing new‐onset AF in hypertensive patients.^79^ Even in the presence of heart failure, statins reduce the prevalence of AF.^80^

### 5.3 Steroids

Given the relationship between inflammation and AF, it would seem reasonable that steroids should help in the prevention or treatment of AF. In fact, studies have supported the use of glucocorticoids in primary and secondary prevention of AF. One study of 88 patients undergoing CABG given methylprednisolone postoperatively showed reductions in onset of AF.^81^ Another study of 138 patients given pulse steroid therapy after catheter ablation found reduced rates of AF.^82^ A higher, single dose of injected methylprednisolone worked better at preventing AF after catheter ablation than did a lower dose in a prospective study of 448 patients.^83^ However, a more recent study of 60 patients undergoing ablation showed no impact on the rate of postprocedural AF despite a reductions in inflammatory markers.^84^ Considering their overall unfavorable side effect profile, the role of steroids in the prevention or treatment of AF is unclear.

### 5.4 Fish oils

The effectiveness of fish oil in the prevention or treatment of AF is controversial. Dietary intake of polyunsaturated fatty acids (PUFAs) improve cardiovascular outcomes, partly through their hypolipidemic effects and partly because of their antioxidant properties.^85^ In fact, the n‐3 fatty acids are often used in the treatment of inflammatory diseases such as rheumatoid arthritis and Crohn's disease. A diet rich in n‐3 fatty acids correlates with reductions in inflammatory markers. The effect on AF is less clear.^86^

In a prospective, randomized, placebo‐controlled study of 1516 patients undergoing cardiac surgery, PUFA did not prove to be protective against the development of AF.^86^ Similarly, another prospective, randomized, placebo‐controlled study of 337 patients with AF found no positive effects on the inflammatory markers and recurrence of AF with the use of fish oils.^87^ A large meta‐analysis of fish oil for prevention of AF in postoperative patients found no effect.^88^ Our conclusions is that fish oil supplementation is unlikely to add benefit in the prevention or treatment of AF.

### 5.5 Vitamin C

Ascorbic acid (vitamin C) is an inexpensive nutritional supplement and is a potent antioxidant. This effect could potentially reduce the inflammation in patients at risk for AF. Vitamin C reduces atrial electrical remodeling, thereby reducing the incidence of AF, possibly through its effects of scavenging reactive oxygen species and reducing overall inflammation.^89^ Studies have shown a modest effect of vitamin C supplementation. In one study of 44 patients undergoing electrical cardioversion, vitamin C was associated with a decrease in AF recurrence.^90^ A recent meta‐analysis by Hemilä et al showed a benefit of vitamin C supplements for the prevention of AF, but only in countries outside of the United States.^91^ Another recent meta‐analysis found that peri‐operative vitamin C reduced the onset of postoperative AF.^92^

## 6 CONCLUSIONS

In summary, there are extensive and expanding evidence showing a connection between inflammation and the development and propagation of AF. These relationships do not adequately define exactly why they are related and further in vitro and in vivo studies can shed further light on the pathophysiology of this connection. Furthermore, several medications have shown protentional beneficial effects in the prevention and reduction of AF. Studies with larger patient population, in particular randomized clinical trials, are needed to better investigate their roles. Newer anti‐inflammatory medications including monoclonal antibodies which demonstrated significant benefit in decreasing events related to atherosclerotic coronary disease could also be the next generation of medications in the AF field and need to be investigated.

## CONFLICT OF INTEREST

None.
